# Differences in affinity of monoclonal and naturally acquired polyclonal antibodies against *Plasmodium falciparum* merozoite antigens

**DOI:** 10.1186/s12866-015-0461-1

**Published:** 2015-07-03

**Authors:** Sreenivasulu B. Reddy, Robin F. Anders, Nadia Cross, Ivo Mueller, Nicolas Senn, Danielle I. Stanisic, Peter M. Siba, Mats Wahlgren, Fred Kironde, James G. Beeson, Kristina E.M. Persson

**Affiliations:** Department of Microbiology, Tumor and Cell Biology, Karolinska Institutet, 171 77 Stockholm, Sweden; Department of Biochemistry, La Trobe University, Vic, 3086 Australia; The Macfarlane Burnet Institute for Medical Research and Public Health, Melbourne, Victoria Australia; The Walter and Eliza Hall Institute of Medical Research, Parkville, Victoria Australia; Papua New Guinea Institute of Medical Research, Goroka, 441 Papua New Guinea; Swiss Tropical and Public Health Institute, Basel, Switzerland; Institute for Glycomics, Griffith University, Queensland, Australia; Department of Biochemistry, Makerere University, Kampala, Uganda; Habib Medical School, IUIU, Kampala, Uganda; Department of Laboratory Medicine, Lund University, 22185 Lund, Sweden

**Keywords:** Antibodies, Antigen, Falciparum, Immunology, Malaria, Parasitology

## Abstract

**Background:**

Malaria is a major global cause of deaths and a vaccine is urgently needed.

**Results:**

We have employed the *P. falciparum* merozoite antigens MSP2-3D7/FC27 and AMA1, used them in ELISA, and coupled them in different ways using surface plasmon resonance (SPR) and estimated affinity (measured as k_d_) of monoclonal as well as naturally-acquired polyclonal antibodies in human plasma. There were major differences in k_d_ depending on how the antigens were immobilized and where the His-tag was placed. For AMA1 we could see correlations with invasion inhibition. Using different immobilizations of proteins in SPR, we could see only moderate correlations with levels of antibodies in ELISA, indicating that in ELISA the proteins were not uniformly bound and that antibodies with many specificities exist in natural immunisation. The correlations between ELISA and SPR were enhanced when only parasite positive samples were included, which may indicate that high affinity antibodies are difficult to maintain over long periods of time. We found higher k_d_ values for MSP2 (indicating lower affinity) compared to AMA1, which might be partly explained by MSP2 being an intrinsically disordered protein, while AMA1 is globular.

**Conclusions:**

For future vaccine studies and for understanding immunity, it is important to consider how to present proteins to the immune system to achieve highest antibody affinities.

**Electronic supplementary material:**

The online version of this article (doi:10.1186/s12866-015-0461-1) contains supplementary material, which is available to authorized users.

## Background

Malaria is a disease that kills up to one million people every year [1]. Immunity against the life-threatening infection caused by *Plasmodium falciparum* develops slowly and only after repeated exposure [2]. Humoral immunity, besides cell-mediated immunity, is important in protection against the disease [3], and it has been shown that antibodies from immune individuals can reduce parasitemia and clear clinical symptoms [4]. Some of the antibodies are directed against merozoite antigens of the parasite, like Merozoite Surface Protein 2 (MSP2) and Apical Membrane Antigen 1 (AMA1) [5–10]. These antigens have both been considered promising vaccine candidates, but so far no vaccine has been shown to be highly efficacious in phase 2 trials [11]. A better understanding of the presentation and form of antigens in vaccines, and the affinity and function of antibodies to these antigens is essential to advance the development of effective vaccines.

**Fig. 1:**
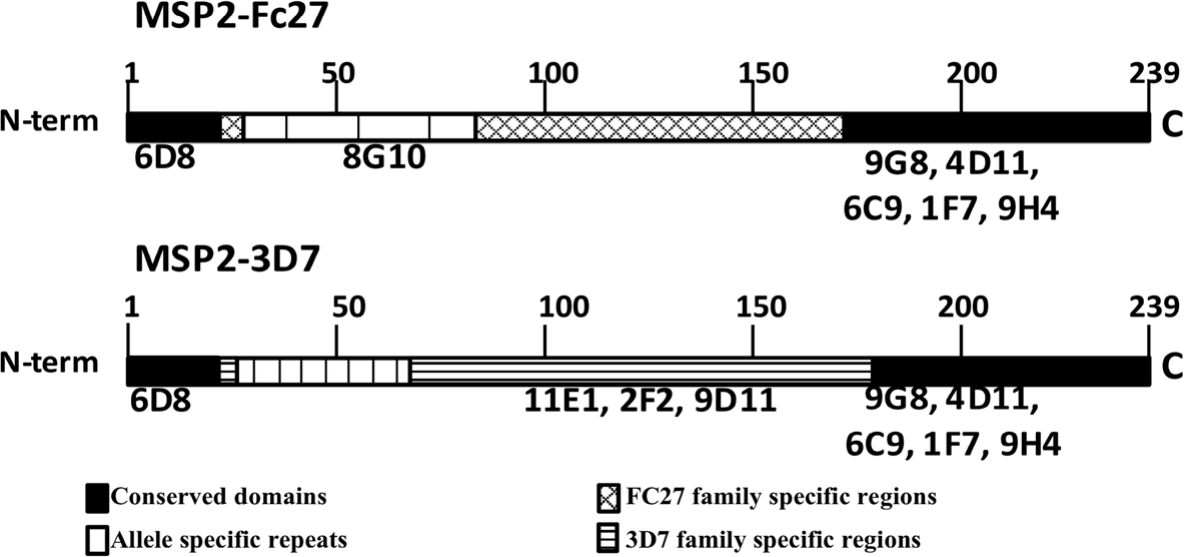
Schematic drawing showing the different mAb binding sites on the MSP2-FC27 and MSP2-3D7 proteins

MSP2 is a 25–30 kDa merozoite membrane protein [12–14], which has a central variable region flanked by conserved N- and C-terminal regions (Fig. 1). MSP2 appears to play an important role in erythrocyte invasion, and is rapidly processed immediately post-invasion [15]. Recombinant MSP2 is an intrinsically disordered protein. However, the parasite antigen appears more ordered, which may be due to interactions with the merozoite membrane or oligomerization of MSP2 [16]. MSP2 parasite alleles can be categorized into two major groups, 3D7 and FC27 [6, 17]. Studies have shown that MSP2 can induce both naturally-acquired and vaccine-induced antibodies that appear to be protective, and that the response is strain-specific [18–20]. A phase 2 trial of a MSP2-containing vaccine demonstrated some protective efficacy [19]. Antibodies raised against MSP2 generally do not directly inhibit invasion [15] but have a growth inhibitory effect in antibody-dependent cellular inhibition (ADCI) assays [14, 21–23].

AMA1 is an 82-kDa type 1 integral membrane protein that is expressed both at the sporozoite- and merozoite stages. Native as well as recombinant forms of the antigen have been shown to induce various levels of protection against challenge from *Plasmodium* parasites in simian [24, 25] and rodent models [26–28]. In humans, presence of naturally-acquired antibodies against AMA1 has been associated with protection from disease [29–33], and monoclonal antibodies have been shown to inhibit invasion of erythrocytes [34–37]. A recent phase 2 trial demonstrated protective effect against malaria due to vaccine-like alleles during the first season after vaccination, but the effect was lost after the second season [38].

Measurement of the affinity or 'functional affinity' [39] of an antibody for its antigen has been shown to be a prominent determinant of the biological efficacy of an antibody [40, 41]. For pathogens such as bacteria, affinity of antibodies has been proven to be of importance for protection from disease after vaccination [42–44]. However, there is limited knowledge of antibody affinity to malaria parasite antigens, how this is influenced by protein structure, how it differs between antigens, and how the presentation of an antigen influences affinity. Moreover, there are limited data defining optimum methods to measure affinity, and the affinity of antibodies directed against unstructured proteins (such as MSP2) in particular. Recently, our group showed that naturally-acquired high affinity antibodies to MSP2-3D7, measured by SPR, were associated with protection against malaria [45].

In this study, we have focussed on conformational changes of the intrinsically disordered MSP2 protein, and studied how these changes result in different binding affinities of naturally-acquired as well as mAb. For comparison, we have also used AMA1, which is a protein with a globular fold and a more stable structure. Different ways of binding a protein to a surface can result in different conformations of the protein, rendering different epitopes accessible to binding of antibodies; this may be especially important for an intrinsically disordered protein like MSP2, We used MSP2 proteins with the His-tag bound to different ends of the proteins for immobilization, to further evaluate antibody binding. These studies are important for optimal selection of vaccine candidates, and for choosing how they should be presented to the immune system, since high affinity antibodies are probably one of the criteria for creating long-term immunity.

## Results

### Estimation of affinity of human antibodies using dissociation rates (k_d_)

For estimation of affinity of antibodies in human plasma, we measured dissociation rates, k_d_, of antibodies in patient plasma. Recombinant antigens were bound using amine N- or C-terminal coupling to CM5 sensor chips, or His-tagged coupling to NTA chips. Plasma was flowed over the chips. One lane was used as reference lane (no antigen present). Swedish non-immune plasma was used to determine the cut-off to be considered as background level. Dissociation rates were independent of plasma concentrations. At least two different plasma concentrations were used to confirm the same values of k_d_ irrespective of concentration. To test the stability of the bound recombinant proteins, a pool of plasma (immune individuals) was used after every 20 samples. The k_d_ values were independent of the loss of protein over time (seen as a decrease in response units). Anti-IgG was added directly after the plasma samples for a subset of ten samples, to confirm the presence of bound IgG to the recombinant proteins (Additional file 1: Figure S1).

**Fig. 2:**
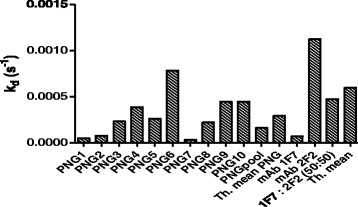
Affinity of antibodies (measured as k_d_) in plasma from individuals living in PNG. MSP2-FC27 was bound to the chip through amine N-terminal coupling. Ten different plasma samples were analyzed separately as well as in a mixture (PNG pool). The pooled sample shows an inclination towards higher affinity, when compared with the theoretically calculated mean (Th. mean) for the same sample mixture

To avoid loosing antibodies that might exist only in low concentrations, and to include antibodies of all affinities, plasma was used without prior purification of antibodies. We evaluated the procedure using mixtures of plasma containing antibodies with different affinities. Ten different PNG plasma samples were analyzed with MSP2-FC27 bound through amine N-terminal coupling. Individual k_d_ ranged from 3.3x10^−5^-7.8x10^−4^ s^−1^ (Fig. 2). The theoretically calculated mean for these samples was 2.9x10^−4^ s^−1^, but when an equal mixture of all ten samples together was measured, the experimental k_d_ was 1.6x10^−4^ s^−1^, indicating a tendency for the mixtures to show a slightly higher affinity than the calculated theoretical one. This was also the case for a combination of mAb; the individual k_d_ for mAb 1 F7 and 2 F2 were 6.8x10^−5^ and 1.1x10^−3^ respectively, whereas the experimental k_d_ for a 50:50-mixture was 4.7x10^−4^ s^−1^.

### Different affinities of human antibodies for different proteins

**Fig. 3:**
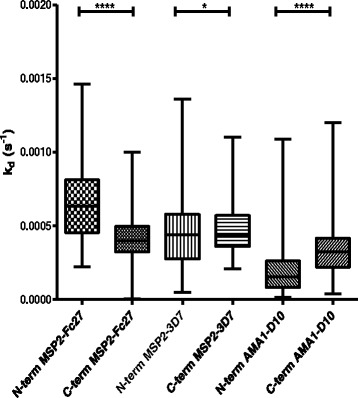
Comparison of affinity responses (measured as k_d_) between amine N- and C- terminal coupling. PNG and Ugandan samples (n = 170) were used together with recombinant MSP2-FC27, MSP2-3D7 and AMA1. The box plot values represent the 25^th^ percentile, median, and the 75^th^ percentile. The whisker range determines the 5th and 95th percentiles

When plasma from individuals in PNG/Uganda (n = 170) were examined using N-terminal amine coupling of antigens, antibodies against AMA1 had the highest affinity, followed by anti-MSP2-3D7 and anti-MSP2-FC27 antibodies (Fig. 3, all p-values < 0.001). Examples of original SPR data is shown in Additional file 1: Figure S2. The results were the same whether PNG or Uganda was analyzed separately, or if all were together (p < 0.0001 for all differences). The same pattern (highest affinity for antibodies against AMA1) was seen for all ages. To establish that the effects of plasma binding to recombinant proteins on the SPR chip was due to antibodies, IgG was purified from 5 of the PNG samples and tested against MSP2-3D7 + MSP2-FC27. There were no significant differences in k_d_ between the purified IgG samples and the corresponding plasma (Additional file 1: Figure S3), indicating that the majority of the effect measured was due to IgG antibodies present in plasma.

The samples from PNG were from asymptomatic individuals. We compared parasite positive and parasite negative individuals, and found that there were no differences in age or affinity between parasite positive and negative individuals, however the number of samples in the parasite positive group was limited.

### Affinity of human antibodies using N-or C-terminal amine coupling of antigens to the chip

After observing variable affinity responses for antibodies against MSP2 and AMA1 proteins, we investigated whether the coupling chemistry and/or orientation of the proteins affected the k_d_ values. Along with using the more common method of N-terminal amine coupling, the recombinant proteins were immobilized onto CM5 chips through a C-terminal amine coupling. Higher k_d_ values for antibodies from Ugandan and PNG samples were observed when C-terminal amine coupling of MSP2-3D7 and AMA1 proteins were used, compared to N-terminal coupling above, whereas the opposite was seen for MSP2-FC27 (Fig. 3). The overall (p < 0.0001) and individual differences in responses between N- and C- terminal couplings were significant (MSP2-FC27/MSP23D7/AMA1; n = 170; p = <0.0001/0.03/0.0001, respectively). When the Ugandan and PNG samples were analyzed separately the results showed similar patterns (PNG; MSP2-FC27/MSP23D7/AMA1; p = 0.003/0.02/<0.0001, respectively; Uganda: MSP2-FC27/MSP23D7/AMA1; p = <0.0001/0.1/0.0002, respectively).

For the PNG samples, there were no differences in the affinities of antibodies to any of the proteins between parasite positive or negative (not shown).

### Affinity of human antibodies using N- or C-terminally His-tagged coupling of antigens

**Fig. 4:**
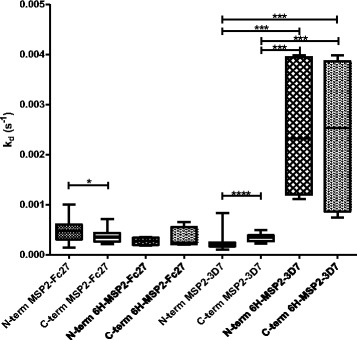
Comparison of affinity response (measured as k_d_) of five PNG plasma samples. Different bindings to the chip for MSP2-3D7 or MSP2-FC27. N-terminal coupling exploits amine groups in the ligand, C-terminal coupling exploits carboxyl groups. Hexa His–N terminal protein coupling uses the capture of His-tagged constructs at the N-terminal end of the protein, and hexa His-C-terminal protein coupling uses the capture of His-tagged constructs at the C-terminal end of the protein. The box plot values represent the 25^th^ percentile, median, and the 75^th^ percentile. The whisker range determines the 5th and 95th percentiles

MSP2-FC27 and MSP2-3D7 were bound to Ni-NTA chips through either N- or C-terminal His-tags. Five different PNG samples were used and flowed over the chips in the same way as for the amine coupling (Fig. 4). We used four different ways of binding MSP2-FC27 (amine N-terminal, amine C-terminal, or through the His-tag at either the N-or C-terminal end), and the only significant difference for binding of the five PNG samples was between amine N- and C-terminal couplings (p = 0.045), with lower affinity for N-terminal coupling. For MSP2-3D7, the different methods of coupling resulted in highly significant differences in antibody-binding affinities (p < 0.0001 for all comparisons, except between N- and C-terminal His-tagged couplings where no difference could be seen). The five samples were from individuals aged 5–9 years, but no obvious trends could be seen with increasing ages (not shown). The most striking finding for the different couplings was the low affinity of antibodies to the His-tagged 3D7 proteins, whether coupled through their N- or C-terminal His-tag.

### Affinity of mAb

**Fig. 5:**
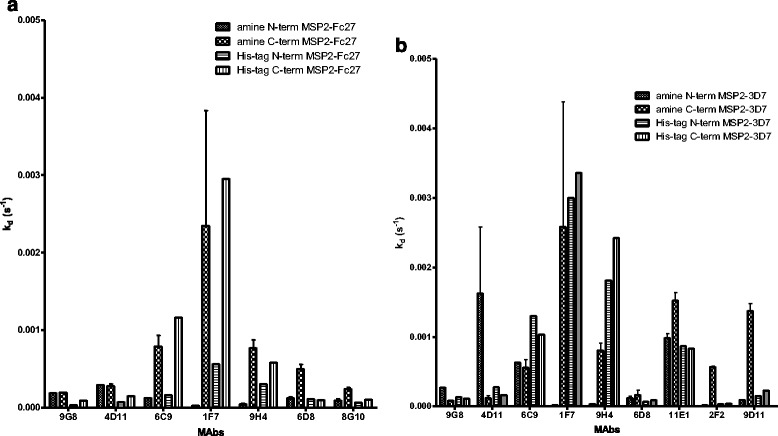
Affinity responses measured as k_d_ values of the mAb against MSP2 using different couplings. The MSP2 proteins (A) MSP2-FC27 (B) MSP2-3D7 were coupled in four different ways to the chip; either through amine or His-tagged N-terminal or C-terminal coupling. The location of mAb binding to MSP2 is shown in Fig. 1

The effect of different coupling procedures on the k_d_ values of anti-MSP2 mAb was examined, with MSP2-FC27/MSP23D7 bound using N- or C-terminal amine coupling and N- or C-terminal His-tag coupling (Fig. 5a, b). Examples of original SPR data is shown in Additional file 1: Figure S2. For MSP2-FC27, a pattern could be discerned between binding of antibodies to different parts of the protein. 9G8 and 4D11, which bind to the conserved C-terminal part of MSP2, generally bound with quite high affinities, but with slightly higher affinities to the His-tagged proteins compared to the amine coupled proteins, and in both cases with similar affinities between N- and C-terminal bindings. 6C9, 1 F7 and 9H4 also bind to the conserved C-terminal part of MSP2, but binding is dependent on an intact disulphide bond, and we could see higher affinities when binding to the N-terminally coupled proteins. 6D8 and 8G10, which bind towards the N-terminal end of MSP2, showed similar, high affinities when using His-tagged coupling but slightly higher affinities when using amine N-terminal coupling compared to amine C-terminal coupling.

For MSP2-3D7, the pattern between different binding sites of the antibodies was less clear. 9G8 and 4D11 (which bind to the conserved C-terminal part of MSP2) bound with lower affinity to the N-terminally amine coupled protein compared to the rest of the proteins. 1 F7 and 9H4 (which also bind to the conserved C-terminal part of MSP2) bound with very high affinity to the N-terminally amine coupled protein, but with lower affinity to the rest of the proteins. 6C9, which is supposed to bind in a similar way as 1 F7 and 9H4, was found to bind with lower affinity for the N-terminally amine coupled protein compared to 1 F7 and 9H4. The mAb 6D8, which recognizes a conserved N-terminal epitope, bound with high affinity whatever way the MSP2-3D7 protein was coupled to the chip. In general, the antibodies that recognized the variable region of MSP2-3D7 (11E1, 2 F2, 9D11) bound with the highest affinity to the protein when using the amine C-terminal coupling.

There was no cross reactivity observed between allele-specific mAb (not shown).

As a comparison to the above MSP2 proteins, we also tested the monoclonal antibody 1 F9, which bound very strongly to AMA1 (coupled through N-termial coupling to the chip). The k_d_ results were in the 10^−6^ range, but as the off-rate was almost like a straight line due to the very strong binding, it was difficult to estimate a true k_d_ value (not shown).

### Levels of antibodies using ELISA and correlations with SPR

All individuals had antibodies in ELISA to the three recombinant antigens studied, but there was no association with age (whether analyzed separately for Uganda/PNG samples or together). For PNG samples there was no difference in antibody levels between parasite positive and negative individuals.

When the antibody levels in ELISA were compared to SPR, correlations were seen for the Ugandan samples (where all had malaria) mainly for AMA1 (ELISA-N-terminal amine coupling R^2^ = 0.25, p < 0.0001, ELISA-C-terminal amine coupling R^2^ = 0.41, p < 0.0001).

**Fig. 6:**
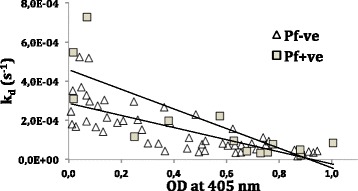
Correlation for PNG samples between ELISA and SPR results for amine N-terminal coupling of AMA1. Parasite positive samples indicated with squares, R^2^ = 0,65 p = 0.0005; parasite negative samples indicated with triangles, R^2^ = 0,57 p < 0.0001; all samples included R^2^ = 0.54 p < 0.0001)

For the PNG samples, there were correlations for AMA1 both when parasite positive and negative samples were included for N-terminal coupling (Fig. 6). For ELISA-C-terminal amine coupling R^2^ = 0.10, p = 0.007 for all samples, R^2^ = 0.54, p = 0.003 only parasite positive and non-significant R^2^ = 0.02 only parasite negative.

Weaker correlations were seen in the PNG samples between ELISA-SPR for MSP2; MSP2-3D7 ELISA-N-terminal amine coupling R^2^ = 0.08, p = 0.02 all samples, R^2^ = 0.31, p = 0.04 only parasite positive. ELISA-C-terminal coupling R^2^ = 0.06, p = 0.04 all samples, R^2^ = 0.34, p = 0.03 only parasite positive. When MSP2-FC27 was used in N-terminal amine coupling, the only correlation with ELISA was for parasite positive (R^2^ = 0.31, p = 0.04).

In conclusion, the analysis showed better correlations between ELISA and SPR results for AMA1 compared to the MSP2 proteins, and better correlations when only parasite positive samples were included in the analysis.

### Invasion Inhibition and correlations with ELISA and SPR

For the Ugandan samples, invasion inhibitory experiments were performed using two clinical isolates (UAS31, UAM37). We could see correlations between invasion inhibition and SPR results for UAM37 and amine N-terminal SPR results for MSP2-3D7 (R^2^ = 0.14, p = 0.03), and for both clinical isolates and amine C-terminal SPR for AMA1 (R^2^ = 0.11, p = 0.01 for UAM37 and R^2^ = 0.08, p = 0.04 for UAS31). For non-significant correlations, R^2^ were <0.03. Invasion inhibition results were also compared to ELISA, and significant correlations were seen for UAS31 versus AMA1 (R^2^ = 0.24, p = 0.0002), UAS31-MSP2-FC27 R^2^ = 0.21, p = 0.0005, and for UAM37 versus AMA1 (R^2^ = 0.21, p = 0.0006). For the non-significant correlations, R^2^ values ranges were <0.10.

## Discussion

For most merozoite surface proteins, the conformation of the native protein located on the surface of the merozoite is not known. A number of proteins, including AMA1, have globular domains stabilized by intramolecular disulphide bonds [26, 29]. Such structures will be relatively stable but protein-protein interactions can result in significant changes in conformation as has been described for AMA1 when it interacts with RON2 [46]. For MSP2, an intrinsically disordered protein, protein-protein interactions, or interactions with the membrane itself, might have major effects on the conformation of the protein and thereby affect the interaction between MSP2 and antibodies. Both AMA1 and MSP2 are known targets of naturally-acquired protective immune responses, as a majority of adults living in malaria endemic regions have high levels of antibodies as measured by ELISA [29, 47, 48]. However, in vaccine studies, it is important to choose proteins that can generate functionally important antibodies. An antibody that is going to function in vivo probably needs to be of high affinity, including a low off-rate, so that once an antibody is bound to the merozoite surface, it can stay there during the invasion process. It has been shown before that it is of importance with both antibody titer and affinity of the antibodies [49, 50]. We tested the monoclonal antibody 1 F9, directed against AMA1, and found an extremely k_d_ value, indicating a very strong binding. This is in line with earlier studies of 1 F9, which have shown that 1 F9 and AMA1 interacts with a very large interface, creating an environment suitable for a stable structure [51]. In future studies, it would be of interest to include other merozoite proteins, such as MSP1, which is also a vaccine candidate, for testing in SPR to investigate whether this protein can also induce high affinity antibodies. The part of MSP1 that is inserted into the erythrocyte consists of two epidermal growth factor-like domains, which (like AMA1) are very stable in their structure due to several disulphide bonds.

Our findings demonstrate that there are major differences in antibody affinity (measured as k_d_) for two leading merozoite vaccine candidates, which may reflect structural differences. We show that the form in which antigens are immobilized significantly influences antibody affinity, which is relevant for understanding how to present antigens in vaccines, and how to measure antibody affinity in vaccine trials and studies of naturally-acquired immunity. Antibody affinity also generally increased with increasing antibody levels, consistent with increasing malaria exposure leading ultimately to protective immunity, including both higher affinity of antibodies as well as higher levels of antibodies. It should be noted that k_d_ values are independent of concentration, which is convenient in this case. In general, measuring antibodies by ELISA cannot be relied on as a good correlate of antibody affinity. It has been shown before when using Guanidine Thiocyanate, that high levels of antibodies against MSP2 is not necessarily related to avidity of the antibodies [52]. Duration of antibodies is also important, MSP2 antibodies have been shown to be of shorter half-life than antibodies against AMA1 [53]. In this case it was probably not because of different antibody subclasses, but in other cases this might be a more important reason for differences in half-lives of antibodies as has been shown for EBA175 [54].

In our studies, we measured k_d_ values of naturally-acquired, human antibodies. We believe that our method is more physiological and more precise than many other methods since we include all antibodies (without purification) in our measurements and the net effect is measured. SPR is also a method where the measurements are performed under flow, which is the natural situation in vivo. Using this method, we could see higher affinity of naturally-acquired antibodies to AMA1 compared to MSP2, and higher affinity for the 3D7 allele of MSP2 compared to FC27. This pattern was consistent whether measured in samples from Uganda or PNG, and for both symptomatic and asymptomatic individuals. The higher affinity for AMA1 could be due to a more stable structure of the protein, making it easier for antibodies to stay bound to their target, compared to MSP2 that has a more flexible structure. For MSP2, strain-specificity has been noted before to be of importance for formation of a protective response [19]. It might be that the 3D7 allele has been more common in the studied populations over the years than FC27; 3D7 is generally more prevalent globally, and is more prevalent in the PNG study population^26^, suggesting that greater exposure may partly explain the greater affinity of antibodies to MSP2-3D7. Also in the Ugandan population, MSP2-3D7 has been shown to be more common with 96 % of patients with uncomplicated malaria being positive, and 74 % being positive for MSP2-FC27 [55]. However, there could also be intrinsic differences in the tendency to form a stable structure between the allelic forms of the proteins. Both forms have central repeat regions that are probably important for formation of antibodies, since many of the antibodies seems to be directed against this part, and not so much against the N- or C- terminal conserved parts [56–59]. However, this central region is different for the two proteins and when we coupled the proteins to the SPR-chips, it was quite clear that there were differences in affinity depending on the way the proteins were bound. For 3D7, the affinity of naturally-acquired antibodies was higher when the protein was bound through its N-terminal part, while for FC27 the affinity was higher when bound through its C-terminal part. These results could be explained by antibodies being directed against epitopes that are central, but still more close to the C-terminal region for MSP2-3D7, and more close to the N-terminal region in MSP2-Fc27 (where the variable regions are positioned).

When the results from SPR were compared to antibody levels measured by ELISA, there were correlations both with results from amine N-terminal and C-terminal coupling, indicating that epitopes at both ends of the protein are available in ELISA. Correlations were often weak, emphasizing that SPR and ELISA are two different methods that measure different things, and correlations might therefore not be consistently present. We also compared SPR results to invasion inhibition assays, and saw very modest correlations. When ELISA was compared to invasion inhibition results, there were stronger correlations between levels of AMA1 and invasion inhibition compared to levels of MSP2, supporting earlier results that have suggested AMA1 antibodies to be directly inhibitory, while MSP2 antibodies might need ADCI to be inhibitory.

For most of the MSP2 mAb, there were clear differences in affinity of bound antibodies depending on which coupling method was used for the proteins. We can’t be sure that what we call “N”-and “C”-terminal coupling is really using those ends of the proteins, but at least we know that it is totally different ways of binding the proteins. The proteins probably form different structures when bound differently, since we know that the mAb are homogenous. It has been shown before that alterations in k_d_ values can be caused by conformational changes in immobilized proteins when SPR has been applied [60–63]. Because this kind of difference was seen both for mAb and for naturally acquired human polyclonal antibodies, we think it is not just an effect of including different antibodies in the polyclonal response, but that antibodies will bind with different affinities depending on how the protein is presented to the antibody. This is important knowledge, since an efficient vaccine should probably be one that induces high affinity antibodies and one has to consider in what way the proteins are presented to the immune system.

For a small number of samples we tried coupling of the MSP2 proteins through their His-tag. Binding of naturally acquired antibodies to MSP2-3D7 gave lower affinity when bound through the His-tag, whether the His-tag was at the N- or C-terminal end (Fig. 4). This might be because the protein itself became more flexible in structure when bound through the His-tag compared to binding with amine coupling, making it more difficult for antibodies to bind. It has been shown before that different environments can affect the structure of MSP2, and it has even been suggested that the N-terminal part of MSP2 can be stabilized by lipid interactions [16]. When mAb were used, the antibodies directed against the conserved C-terminal part of MSP2 also showed a lower affinity towards the His-tag coupled proteins. Since this pattern was only seen when mAb against the conserved C-terminal part of the protein were used, one can speculate that many of the antibodies in plasma were also directed against this part, at least for MSP2-3D7. These results point out the potential importance of the exact placement of added tags for creating a more stable structure in proteins, especially when using intrinsically disordered proteins, in forming binding sites for functionally important antibodies. In most SPR studies, N-terminal coupling of proteins is used, since this is the easiest to perform. C-terminal coupling includes an extra step of serial dilutions of PDEA, and when His-tags are used to bind proteins to the chips, every regeneration induces total loss of the bound protein from the chips, which makes the experiments much more cumbersome to perform. However, one should be aware of possible differences in results that can be induced through different forms of binding of proteins, when using SPR. The structure of MSP2 in the merozoite is not known, and part of the reason for that is probably that it is an unstructured protein, which makes it more challenging to study compared to a protein with a more stable structure, but placing His-tags at different ends might help in facilitating for some specific antibodies to bind. However, further studies are needed to clarify the exact structure of MSP2 on the surface of the merozoite, and when this is known it should be easier to decide which antibodies are best in protecting patients from malaria.

## Conclusions

In conclusion, we have measured affinity of both monoclonal and naturally-acquired antibodies in human plasma, and we showed that the measurement of affinity was dependent on how the proteins were bound to surfaces and presented, and in which way the tag was bound to the protein. This information is crucial for vaccine studies, where the goal is to have the best possible conformation of a protein and expose important epitopes to create antibodies that are of high affinity and that can be functional over a long period of time.

## Methods

### Plasma samples

Plasma samples were collected from residents of Madang Province, Papua New Guinea (PNG). The samples were from a cross-sectional bleed in 2007 from individuals 1–52 years, of which 22 % were from parasite positive individuals (determined by microscopy). This population experiences year-round transmission of Plasmodium falciparum malaria with seasonal variation. 100 PNG samples were screened for IgG to MSP2 by ELISA. Positive samples (n = 74) were used. The Ugandan samples (n = 96) were collected in a cohort study [64] from children 6 months - five years of age (mean age 20 months) with malaria. The area of Apac has one of the world’s highest transmission rates of malaria: EIR 1500/year.

### Proteins and monoclonal antibodies

Recombinant MSP2-FC27/MSP2-3D7 proteins with hexa-His tags either at N- or C-termini and AMA1-D10 were expressed in E. coli and purified as described [12, 65]. The MSP2 proteins that were used for amine coupling in SPR analysis had the His-tag at the N-terminal end. SDS-PAGE confirmed recombinant proteins to be monomers (not shown). The mAb against MSP2 were produced in mice [14]. 6D8 is directed against the conserved N-terminal region of MSP2 3D7/FC27. 11E1, 9D11, 2 F2 are directed against the MSP2-3D7 variable region, 8G10 against the MSP2-FC27 variable region. 1 F7, 6C9, 9H4, 4D11 and 9G8 are directed against the conserved C-terminal regions in MSP2 3D7/FC27. All mAbs were diluted in HBS-EP running buffer.

### Coupling of recombinant proteins to SPR chips

The different protein antigens were immobilized onto the sensor chip surface as described in the manufacturers’ protocol for Biacore. For N-terminal coupling, a CM5 sensor chip was used together with an N- terminal amine coupling kit from Biacore, using EDC/NHS chemistry [66]. The carboxymethylated dextran surface of the chip was activated using an injection pulse (10 min, 5 μL/min) containing a mixture of 0.05 M NHS and 0.05 M EDC. The protein immobilization was accomplished by injecting the protein solution at 100 μg mL^−1^ in coating buffer (0.01 M sodium acetate buffer, pH 4.0) until the desired response units were achieved. The remaining sites on the sensor surface were blocked by injecting 1 M ethanolamine (pH 8.5) for 10 minutes. All steps were carried out in a continuous flow of HBS-EP running buffer at 5 μL/min, and all buffers were degassed prior to use.

The C- terminal coupling of proteins was achieved by serial dilutions of PDEA in 0.1 M 2-morpholinoethanesulphonic acid (MES), pH 5.0 (final concentration 4.0 mM - 0.5 mM) and a constant concentration of protein (4.0 μg/mL) and EDC (0.02 M), resulting in an introduction of reactive disulfide groups on carboxyl residues in the protein. The mixture was allowed to react for 1 hour on ice. The following was injected into the SPR machine in sequence; 30 μL NHS/EDC mixture; 20 μL 40 mM cystamine dihydrochloride in 0.1 M borate buffer pH 8.5; and 20 μL 0.1 M dithioerythritol (DTE) in 0.1 M borate buffer pH 8.5. Finally, the protein was bound by injecting the protein-PDEA mixture. Starting with the tube having the least PDEA, the protein solution was injected until desirable response units were achieved. To block the unbound sites, 20 μL 20 mM PDEA in 0.1 M formate buffer pH 4.3 containing 1 M NaCl, was injected.

Immobilization of histidine-tagged MSP2-FC27 and MSP2-3D7 proteins (N- or C- terminal) through the His-tag to a sensor chip NTA was achieved using the manufacturer’s protocol [67]. First, the sensor chip was loaded with Ni^2+^ using a one minute pulse of 500 μM NiCl_2_ in modified running buffer (10 mM HEPES, 150 mM NaCl, 50 μM EDTA. 0.005 % Surfactant P20, pH 7.4), followed by an injection with the His-tagged protein (200 nM). Finally, a one minute pulse of NiCl_2_ (500 μM) was injected to saturate any remaining NTA sites with Ni^2+^ ions (40 – 50 RU), and further increase in NiCl_2_ concentration had no effect on the immobilization level. After analyzing the binding interactions with respective ligands, the chip was treated with a three minute pulse of regeneration solution (10 mM HEPES, 150 mM NaCl, 350 mM EDTA, 0.005 % Surfactant P20, pH 8.3), which stripped all the His-tagged proteins and Nickel off the chip, demonstrated by the lack of binding after injection with the same His-tagged proteins.

In conclusion, N-and C-terminal binding of proteins means covalent binding of proteins to the sensor chip surface, while when the His-tag is used for binding, the binding is achieved through the Nickel-His binding. N-terminal binding uses amine coupling, and since there is an easily accessible amine group at the N-terminal end of all proteins, this end of the protein will be bound to the chip. However, also other amine groups in the protein could potentially bind to the chip, depending on how accessible the amine groups are. The same reasoning can be applied to C-terminal coupling. Even if we can not be 100 % sure that what we call N-and C-terminal coupling is always really N-and C-terminal coupling, at least we can be sure that the couplings are different, and we think that quite often the bindings are actually through the ends of the proteins. When the His-tag is used to couple the protein, there are no other options for the protein but to use the His-tag to bind to the chip.

### Binding assays and analysis

The SPR binding assays were performed with a constant flow rate of 30 μl min^−1^ at 25 °C. Plasma samples and purified monoclonal antibodies (in different dilutions, 1:7.5, 1:15, 1:30, 1:60 or 1:90) were flowed over the bound recombinant proteins in HBS-EP buffer, pH 7.4. At all dilutions, the association of antibodies with the immobilized MSP2-FC27, MSP2-3D7 and AMA1 proteins was monitored for 3 minutes followed by 10 minutes of dissociation. Residual bound antibody was removed by washing the chip with 10 mM glycine-HCl (pH 1.5) for 5 seconds at 5 μL/min. Reequilibration between the sensor surfaces and running buffer was established prior to injection of the next sample. Response was monitored as a function of time at 25 °C. Kinetic parameters were evaluated by using the BIAevaluation 4.1 software.

### ELISA

ELISA plates (Maxisorb; NUNC 44-2404-21; Denmark) were coated overnight with 200 ng per well of purified recombinant proteins (MSP2-FC27, MSP2-3D7 or AMA1) in PBS, blocked with PBS–5 % skimmed milk powder, and used for standard ELISA [33]. After washing 3x with 0.1 % Tween/PBS, plasma samples were added in different dilutions. After another washing step, rabbit anti-human IgG conjugated to alkaline phosphatase (Sigma, A9544) was applied as a detection antibody. Finally, *p*-nitrophenyl phosphate tablets (Sigma, N2765) was used as a substrate. Color development was measured as the OD at 405 nm. All ELISAs were done in triplicates.

### Invasion Inhibition Assay

The method to study invasion inhibitory antibodies has been described previously [68]. Two Ugandan *P. falciparum* isolates [64], UAM37 (from a patient with mild malaria, containing both FC27 and 3D7 alleles of MSP2), and UAS31 (from a patients with severe malaria, containing the 3D7 allele of MSP2) were cultured *in vitro* (for a couple of months after collection from the patients) in AB+ non-immune Swedish serum and gassed with 90 % NO2, 5 % O2 and 5 % CO2 and placed in a shaker incubator. In brief, parasites were synchronized (5 % sorbitol, v/w) before assay start, and at the day of the assay the majority of the parasites were at late-pigmented trophozoite stage. 50 μl of parasite suspensions were cultured for one cycle in 96 well plates. 5 μl of dialyzed test plasma was added to each well and all samples were run in duplicate. Plates were incubated in a sealed, humidified, gassed box and put in an incubator for 48 hours at 37 °C. Parasitemia was estimated using hydroethidine (10 ug/ml; Sigma Aldrich) in a flow cytometer (FACS Scan; BD) after approximately 48 hours (determined by the parasite stage). Parasite invasion for each sample was measured in comparison to controls (invasion in presence of dialyzed non-immune plasma).

### Statistics

Data analyses were performed using GraphPad Prisma Version 5.0a software. To test for individual differences in affinity to the recombinant antigens, Mann–Whitney test was used. The Pearson correlation coefficient was used to assess the association between invasion/ELISA/SPR. P-values were considered significant if they were <0.05. For collectively comparing the different recombinant proteins, Annova by Kruskal-Wallis was used.

### Ethical Approval

Written informed consent was obtained from all participants or from their guardians, ethical permission number 03–095 (Regionala Forskningsetikkomittén, KI, Stockholm) and MV717 from Uganda National Council for Science and Technology.

## Additional file

Additional file 1: Figure S1. Original SPR data where AMA1 was bound to the chip (using N-terminal coupling). First plasma from one individual living in PNG was injected over the chip, and antibodies from plasma bound to AMA1. At around 200 seconds, buffer was flowed over the chip to view the dissociation phase of the antibodies in plasma from AMA1. At around 400 seconds, anti-IgG (Fig 1A) or anti-IgM (Figure 1B) was injected until around 600 seconds, where buffer was again flowed over the chip. For these data, it was not possible to calculate a kd value, as we could not be sure whether the dissociation was due to only Ig, or both anti-Ig and Ig coming off the chip. But from looking at the graphs in the example shown, we can see a clear binding of anti-IgG but no binding of anti-IgM. **Figure S2.** Original SPR data showing binding of antibodies in plasma from an individual living in PNG, binding to N-terminally coupled AMA1 (top curve), MSP2-3D7 (middle curve), MSP2-FC27 (bottom curve). Plasma was flowed over the chip for 3 minutes (association phase) and dissociation was measured for 10 minutes. **Figure S3.** Original SPR data showing the monoclonal antibodies 6D8 (top curve), 9H4 (middle curve) and 11E1 (bottom curve) binding to MSP2-FC27 (N-terminally coupled to the chip). 11E1 is specific for MSP2-3D7 and does not bind to MSP2-FC27. The antibodies were flowed over the chip for 3 minutes (association phase) and dissociation was measured for 10 minutes.
