# Supplementary material for: Differences in affinity of monoclonal and naturally acquired polyclonal antibodies against Plasmodium falciparum merozoite antigens
Source: BMC Microbiol. 2015 Jul 3;15:133. doi: 10.1186/s12866-015-0461-1 (PMC4491891; doi:10.1186/s12866-015-0461-1)

Supplementary Figures

Supplementary Figure 1.

Original SPR data where AMA1 was bound to the chip (using N-terminal coupling). First plasma from one individual living in PNG was injected over the chip, and antibodies from plasma bound to AMA1. At around 200 seconds, buffer was flowed over the chip to view the dissociation phase of the antibodies in plasma from AMA1. At around 400 seconds, anti-IgG (Fig 1A) or anti-IgM (Figure 1B) was injected until around 600 seconds, where buffer was again flowed over the chip. For these data, it was not possible to calculate a k_d value, as we could not be sure whether the dissociation was due to only Ig, or both anti-Ig and Ig coming off the chip. But from looking at the graphs in the example shown, we can see a clear binding of anti-IgG but no binding of anti-IgM.

Supplementary Figure 2.

Original SPR data showing binding of antibodies in plasma from an individual living in PNG, binding to N-terminally coupled AMA1 (top curve), MSP2-3D7 (middle curve), MSP2-FC27 (bottom curve). Plasma was flowed over the chip for 3 minutes (association phase) and dissociation was measured for 10 minutes.

Supplementary Figure 3

Original SPR data showing the monoclonal antibodies 6D8 (top curve), 9H4 (middle curve) and 11E1 (bottom curve) binding to MSP2-FC27 (N-terminally coupled to the chip). 11E1 is specific for MSP2-3D7 and does not bind to MSP2-FC27. The antibodies were flowed over the chip for 3 minutes (association phase) and dissociation was measured for 10 minutes.

Supplementary Figure 1A

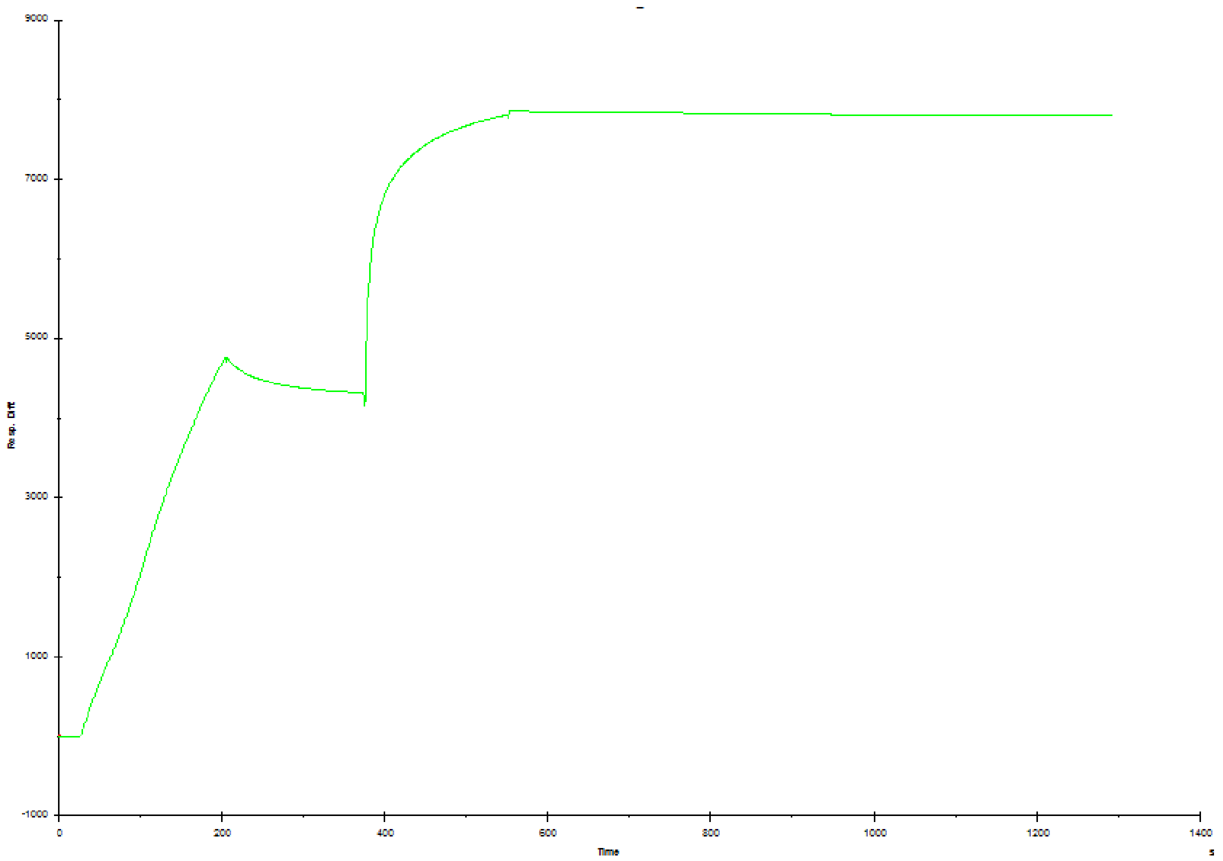

Supplementary Figure 1B

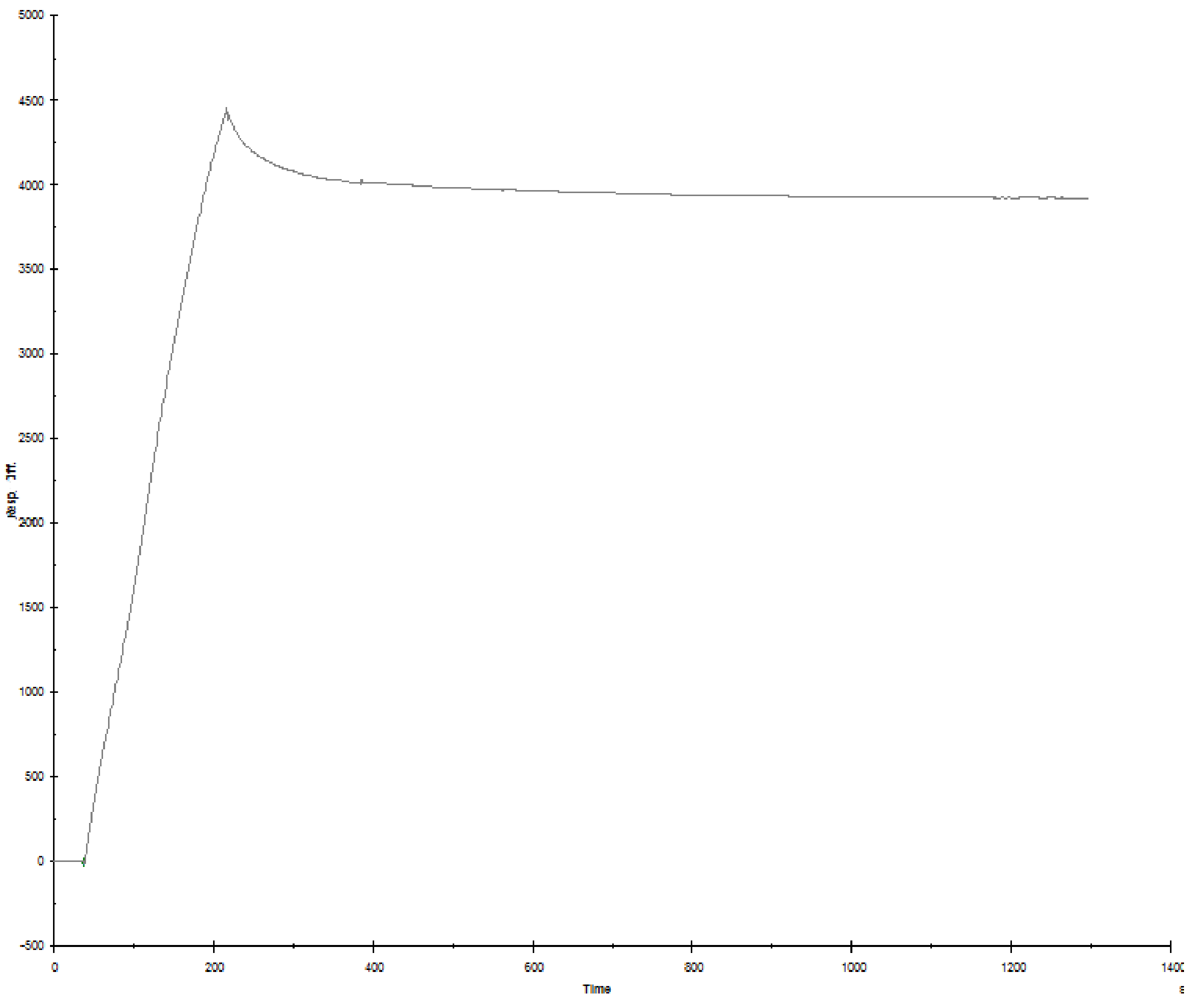

Supplementary Figure 2

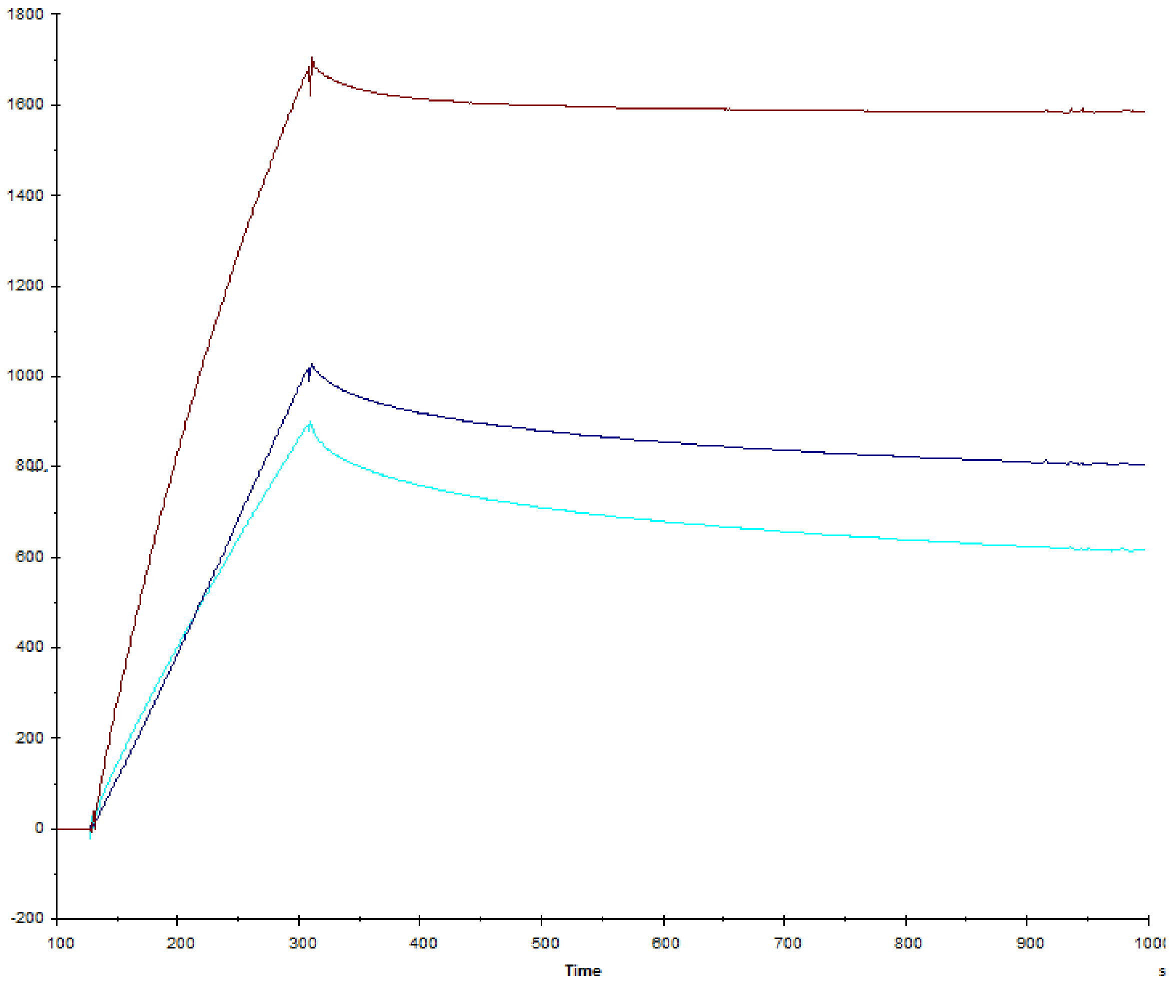

Supplementary Figure 3

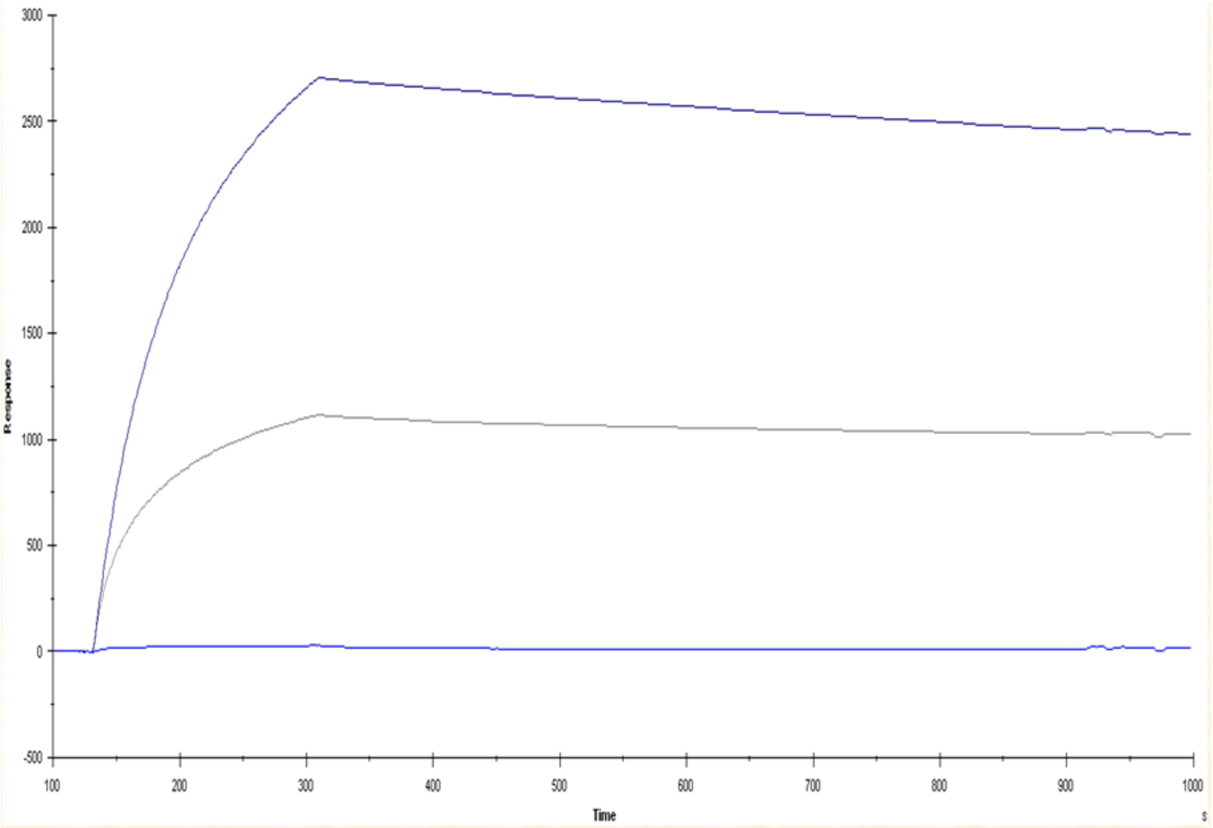

Supplement: Additional file 1: Figure S1. — Original SPR data where AMA1 was bound to the chip (using N-terminal coupling). First plasma from one individual living in PNG was injected over the chip, and antibodies from plasma bound to AMA1. At around 200 seconds, buffer was flowed over the chip to view the dissociation phase of the antibodies in plasma from AMA1. At around 400 seconds, anti-IgG (Fig 1A) or anti-IgM (Figure 1B) was injected until around 600 seconds, where buffer was again flowed over the chip. For these data, it was not possible to calculate a kd value, as we could not be sure whether the dissociation was due to only Ig, or both anti-Ig and Ig coming off the chip. But from looking at the graphs in the example shown, we can see a clear binding of anti-IgG but no binding of anti-IgM. Figure S2. Original SPR data showing binding of antibodies in plasma from an individual living in PNG, binding to N-terminally coupled AMA1 (top curve), MSP2-3D7 (middle curve), MSP2-FC27 (bottom curve). Plasma was flowed over the chip for 3 minutes (association phase) and dissociation was measured for 10 minutes. Figure S3. Original SPR data showing the monoclonal antibodies 6D8 (top curve), 9H4 (middle curve) and 11E1 (bottom curve) binding to MSP2-FC27 (N-terminally coupled to the chip). 11E1 is specific for MSP2-3D7 and does not bind to MSP2-FC27. The antibodies were flowed over the chip for 3 minutes (association phase) and dissociation was measured for 10 minutes. [file 12866_2015_461_MOESM1_ESM.pdf]
